# At the Intersection of Psychiatry and Cardiology: Assessment of Depressive, Anxiety and Cognitive Disorders in Patients Before and After the Implantation of Cardiac Implantable Electronic Devices

**DOI:** 10.3390/medicina62071255

**Published:** 2026-06-29

**Authors:** Kamila Klimek-Ociepka, Karolina Kruczaj, Maciej Dyrbuś, Robert Pudlo, Mariusz Gąsior, Mateusz Tajstra

**Affiliations:** 1 Student’s Scientific Society, 3rd Department of Cardiology, School of Medical Sciences in Zabrze, Medical University of Silesia, 41-800 Zabrze, Poland; 2 3rd Department of Cardiology, School of Medical Sciences in Zabrze, Medical University of Silesia, 41-800 Zabrze, Polandmtajstra@sum.edu.pl (M.T.); 3 Department and Clinic of Psychiatry, Faculty of Medical Sciences in Zabrze, Medical University of Silesia, 42-600 Tarnowskie Góry, Poland

**Keywords:** cardiac implantable electronic devices, implantable cardioverter-defibrillator, cognitive impairment, depression, anxiety, psychological assessment

## Abstract

*Background and Objectives:* Cardiac implantable electronic devices (CIEDs), including permanent pacemakers, implantable cardioverter-defibrillators (ICDs), and cardiac resynchronization therapy (CRT) devices, are increasingly used in an aging population characterized by multimorbidity and a high prevalence of psychiatric and cognitive disorders. Depression, anxiety, post-traumatic stress disorder (PTSD), mild cognitive impairment, and dementia may affect informed consent, adherence to therapy, quality of life, and long-term cardiovascular outcomes in patients undergoing CIED implantation. The aim of this review was to summarize the prevalence and clinical significance of psychiatric and cognitive disorders in patients undergoing CIED implantation and to discuss practical strategies for their assessment in routine cardiology practice.* Materials and Methods:* This narrative review was based on a literature search of PubMed, Web of Science, and the National Health Library database covering the literature from 2010 to 2025, including a broad search strategy. Original studies and review articles were included, having confirmed their definite association with the subject. *Results:* Psychiatric disorders and cognitive impairment were highly prevalent among patients with CIEDs and were associated with impaired adherence, reduced participation in rehabilitation, lower quality of life, and worse cardiovascular outcomes. ICD therapies, particularly inappropriate therapies, were strongly associated with anxiety, depressive symptoms, and PTSD-related distress. Cognitive impairment may compromise informed consent, recognition of complications, and compliance with post-implantation recommendations. Several validated screening tools, including MMSE, MoCA, HADS, BDI-II, STAI, and FSAS, may facilitate early identification of high-risk patients, and their most appropriate use in various clinical scenarios, including pre- and post-CIED implantation was described. *Conclusions:* Psychiatric disorders and cognitive impairment are common and clinically relevant in patients undergoing CIED implantation. Routine psychological and cognitive assessment before and after implantation should be considered an important component of modern multidisciplinary cardiac care, potentially affecting the decision to implant the device, and likely influencing the type of the implanted device.

## 1. Introduction

The use of cardiac implantable electronic devices (CIEDs), including permanent pacemakers (PMs), implantable cardioverter-defibrillators (ICDs), and cardiac resynchronization therapy (CRT) devices, has expanded substantially owing to growing evidence supporting their efficacy and safety across a broad range of cardiovascular indications. As populations age and the prevalence of cardiovascular disease continues to rise, the number of patients undergoing CIED implantation is increasing worldwide. Importantly, the majority of these patients are older adults, with the typical age at implantation frequently exceeding 70 years [1]. This demographic shift has important clinical implications. The prevalence of heart failure (HF), conduction disturbances, atrial arrhythmias, and sudden cardiac death risk all increase significantly with age, making elderly patients the predominant population receiving device-based therapy. In particular, HF affects nearly 20% of individuals over 75 years of age [2], contributing to the growing number of patients undergoing ICD, or CRT implantation, mostly in this patient group. At the same time, advancing age is associated with increasing multimorbidity, frailty, polypharmacy, and a higher risk of drug–drug interactions, all of which may influence both procedural outcomes and long-term prognosis [3].

Despite remarkable technological progress in device therapy, including miniaturization of the devices, and development of completely new strategies for delivering pacing, and defibrillation, psychological and cognitive comorbidities remain under-recognized determinants of outcomes in patients undergoing CIED implantation. Mental health disorders, particularly depression, anxiety disorders, post-traumatic stress disorder (PTSD), cognitive impairment, and dementia, are highly prevalent in this population and may significantly affect adherence to therapy, participation in rehabilitation, quality of life, and even long-term cardiovascular outcomes [4]. In patients with ICDs, fear of device shocks may become a major source of psychological distress, while in most elderly device recipients, baseline cognitive dysfunction may substantially complicate obtaining informed consent, follow-up, and self-management. Importantly, the relationship between cardiovascular disease and psychiatric disorders is bidirectional [4,5,6]. Psychiatric and cognitive disorders are common in patients receiving CIEDs and may adversely affect adherence, quality of life, and clinical outcomes. Given these mechanisms, psychiatric and cognitive assessment should be considered an important part of the care pathway before and after CIED implantation [4,5]. Early recognition of mental health disorders may improve patient selection, optimize informed consent, individualize follow-up, and improve both patient-reported and clinical outcomes.

The aim of this review is to characterize the most prevalent psychiatric and cognitive disorders in patients undergoing CIED implantation, discuss their clinical implications before and after device implantation, and present practical screening strategies that may be incorporated into routine cardiology practice.

### Mental Health and Cognitive Disorders, and Their Risk Factors, in Patients Being Assessed for and Following CIED Implantation

The prevalence of both cardiovascular disease and psychiatric disorders increases with advancing age. Among older adults, depressive disorders affect approximately 26% of women and 19% of men [6]. Their etiology is multifactorial and includes age-related structural and functional changes within the central nervous system, multimorbidity, chronic pain, and reduced functional independence [7]. Depression has been associated with poorer adherence to pharmacotherapy, reduced participation in cardiac rehabilitation, missed follow-up visits, and lower compliance with medical recommendations [8]. This interaction is also relevant in patients undergoing CIED implantation, as such factors may directly influence the effectiveness and safety of device therapy. Despite its clinical importance, depression remains underdiagnosed throughout the continuum of cardiovascular care, partly because symptoms may overlap with manifestations of chronic heart disease, frailty, or aging itself [6]. Although formal diagnostic criteria based on ICD-11 and DSM-5 classifications are available, their complexity may limit their use outside psychiatric settings [9]. Consequently, brief screening tools should play a larger role in routine cardiology practice.

Cognitive disorders represent another major challenge in this population. Dementia affects nearly 5% of the global population and its prevalence rises sharply with age, affecting approximately 12% of individuals aged 60–65 years, with the risk doubling every five years thereafter [4,6]. Alzheimer’s disease remains the most common cause, accounting for approximately 60–80% of cases [10]. Cognitive decline may significantly impair a patient’s ability to provide informed consent, recognize symptoms, comply with treatment, and respond appropriately to device-related alerts or complications. Milder forms of impairment, such as mild cognitive impairment (MCI), are also highly relevant, particularly in elderly patients. Although these individuals may remain functionally independent, MCI is associated with a high risk of progression to dementia and may influence long-term device management [11].

An important limitation of the current evidence base is that patients with pre-existing psychiatric disorders are frequently underrepresented in prospective cardiovascular studies and are less likely to attend screening and follow-up visits. Consequently, this population remains insufficiently characterized in the literature. Furthermore, mental health is still often underemphasized in cardiovascular prevention guidelines, and currently used cardiovascular risk prediction models may underestimate the true risk profile in patients with psychiatric comorbidities [5,12].

## 2. Materials and Methods

**Figure 1:**
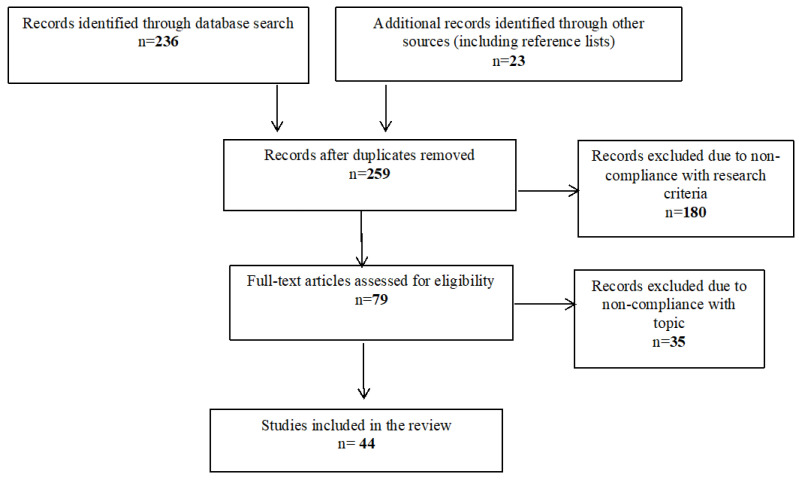
Study inclusion flowchart.

This study was designed as a narrative review of the literature. The literature search covered publications from 2010 to 2025 and was conducted using PubMed, the National Health Library journal database, and Web of Science. The search strategy included keywords in both English and Polish, as well as their combinations, including: “cardiac implantable electronic devices”, “pacemaker”, “implantable cardioverter-defibrillator”, “cardiac resynchronization therapy”, “depression”, “anxiety”, “post-traumatic stress disorder”, “PTSD”, “dementia”, “psychological assessment”, and “cardiac device”. Full-text review articles and original studies were considered eligible for inclusion. Exclusion criteria comprised case reports, conference abstracts without full-text availability, experimental studies involving cell or animal models, and studies conducted in pediatric populations. Following the selection process, 44 references were included, comprising original research articles, review papers, book chapters, and one doctoral thesis. The primary focus of the review was on anxiety, depression, PTSD, and cognitive disorders in patients undergoing CIED implantation. Additionally, factors influencing quality of life and available psychological assessment tools in this patient population were also evaluated. The flow diagram (Figure 1) is provided to illustrate the selection process.

## 3. Clinical Implications of Psychiatric and Cognitive Disorders in Patients with CIEDs

The standard qualification process for CIED implantation typically includes confirmation of the cardiac indication, general clinical assessment, laboratory testing, and appropriate imaging studies [5,13]. However, psychiatric and cognitive evaluation before implantation is also very important, particularly in elderly and frail patients, in whom baseline mental health status may substantially influence informed consent, adherence to follow-up, and long-term clinical outcomes. Several studies suggest that CIED implantation may be associated not only with improved cardiac performance but also with better cognitive function and enhanced quality of life in selected patient groups [14,15,16,17,18,19,20]. Studies demonstrated improvement and stabilization in cognitive performance at 3–24 months after CRT implantation [16,21]. Although causality cannot be definitively established, these observations are biologically plausible and may be explained by improved left ventricular systolic function, increased cardiac output, and improved cerebral perfusion, most likely in responders to CRT. A similar phenomenon has been reported in patients undergoing pacing for symptomatic sinus bradycardia, again, most likely due to improved chronotropic support leading to increased cerebral blood flow. Conversely, although such studies are lacking, it is conceivable that patients who develop pacing-induced cardiomyopathy may experience secondary cognitive deterioration due to worsening cardiac output and systemic congestion, although direct evidence for this fact is currently lacking.

Data regarding cognitive outcomes after ICD implantation remain inconsistent, with some studies suggesting cognitive decline following ICD therapy, whereas others have reported stable cognitive performance even among patients experiencing device shocks [22,23]. Given the difficulty in distinguishing the effects of ICD therapy itself from the neurological consequences of the underlying arrhythmic event or cardiac arrest, these findings should be interpreted with caution. On the other hand, the relatively low incidence of clinically overt stroke following ICD shocks suggests that potential shock-related embolic phenomena, if present, may result in clinically silent cerebral microembolization rather than manifest ischemic stroke, potentially contributing to progressive cognitive decline over time [24]. However, data on this subject remain scarce. Nevertheless, the potential risk of cognitive deterioration should not be overlooked, particularly in patients with pre-existing vulnerability to cognitive decline. In such cases, adequate education of both patients and their caregivers is important, and particular care should be taken to ascertain that consent for device implantation is fully informed.

Anxiety and depressive symptoms are common among patients receiving ICDs and CRT-D devices and may significantly affect quality of life, psychosocial functioning, and adaptation to device therapy [24,25,26,27,28,29,30,31]. In particular, ICD therapies, especially inappropriate or recurrent shocks, may increase emotional distress and contribute to persistent fear of future shocks, highlighting the importance of routine psychological assessment and patient education.

### 3.1. Cognitive Impairment and Practical Implications for CIED Management

Cardiovascular disease and cognitive impairment share several common pathophysiological mechanisms. Preserving normal cerebral function usually requires adequate brain perfusion. In patients with HF, reduced cardiac output may lead to chronic cerebral hypoperfusion, potentially resulting in microstructural injury, clinically manifesting as cognitive decline [32]. The initial symptoms of mild cognitive impairment (MCI) are often subtle and may be overlooked or downplayed by both patients and clinicians. For this reason, a reliable assessment should extend beyond the patient interview and include information obtained from family members, caregivers, or other individuals who have regular daily contact with the patient. They are frequently the first to notice changes in memory, behavior, executive functioning, or daily performance [11]. This approach is particularly important in view of the risk of progression from MCI to the clinically overt dementia, including Alzheimer’s disease, which has been estimated to range from 6% to as high as 30% per year, depending on the studied population [11]. In patients with CIEDs, the type of implanted device may influence cognitive outcomes. As discussed above, patients undergoing CRT implantation may experience improvement in cognitive performance, most likely secondary to enhanced left ventricular systolic function and improved cerebral perfusion. Comparable findings have not been consistently reported in recipients of non-CRT devices [13,33].

The most widely used tools for the assessment of cognitive function in routine clinical practice include the Mini-Mental State Examination (MMSE) and the Montreal Cognitive Assessment (MoCA) [34,35]. MMSE and MoCA are the most commonly used screening tools (Table 1), with the former being a rapid and widely used screening instrument for global cognitive assessment, particularly useful for identifying and monitoring established cognitive impairment and dementia [34,35]. The MoCA evaluates a broader range of cognitive domains and is generally regarded as more sensitive for detecting mild cognitive impairment and early cognitive decline [33,34,35,36].

Assessment of cognitive function in patients eligible for CIED implantation has important practical implications that extend beyond the ability to provide informed consent. Cognitive deficits may significantly impair adherence to perioperative and postoperative recommendations, including restrictions on upper limb movement during the first weeks after implantation, wound care instructions, and the need to avoid manipulation of the device pocket. Failure to understand or comply with these instructions may increase the risk of local complications such as pocket hematoma, wound dehiscence, and device-related infection, as well as rare but clinically significant mechanical complications, including Twiddler’s syndrome and Reel syndrome [37]. Patients with cognitive impairment may fail to recognize or promptly report symptoms suggestive of early post-implantation complications, such as pain, hematoma, swelling of the wound site, or fever. This may delay the diagnosis and treatment of CIED-related infection and other early complications. Early identification of cognitive deficits therefore enables the implementation of additional safety measures, including caregiver involvement, intensified education of both the patient and the family, and individualized modification of the post-implantation follow-up schedule. Such strategies may contribute to reduced complication rates and improved overall safety of device therapy.

Finally, an important consideration in patients with cognitive impairment is the choice of device type. Leadless pacemakers may represent a particularly attractive option in selected patients, as they eliminate the need for a subcutaneous pocket and transvenous leads [38,39,40]. This may reduce the risk of device manipulation and associated complications such as aforementioned Twiddler’s syndrome, which are more likely to occur in individuals with cognitive dysfunction, anxiety, or behavioral disorders. In addition, the absence of a visible or palpable device may decrease psychological distress in some patients. Therefore, in patients at increased risk of non-adherence, pocket self-manipulation, or device-related anxiety, leadless pacing systems may be considered as part of an individualized treatment strategy. It should be emphasized, however, that direct evidence supporting the superiority of leadless pacemakers over conventional devices in patients with cognitive impairment is currently lacking.

### 3.2. Anxiety

Anxiety may be highly prevalent among patients with cardiovascular disease, including those with arrhythmias, heart failure, and subjects treated with CIEDs. Across published studies, clinically relevant symptoms of anxiety and depression have been reported in approximately 20% to 44% of these patients [4,7,25,30,31]. In recent years, the concept of heart-focused anxiety (HFA) has gained increasing attention, referring to excessive anxiety related specifically to cardiac sensations, symptoms, and anticipated cardiovascular events. Those may include, e.g., anxiety related to the occurrence of malignant arrhythmias or heart failure decompensation. HFA may characterize as an elevated vigilance toward catastrophic interpretation of bodily sensations, and avoidance of any activities perceived as potential triggers of cardiac events [17]. Among patients with CIEDs, HFA is particularly pronounced in patients with an ICD. In this group, anxiety most commonly relates to the fear of device high-voltage therapy and the anticipated pain associated with the shock [31,41]. As a consequence, ICD recipients may experience a substantial psychological burden, which in turn may significantly impair their quality of life. The most frequently observed behavioral consequence is avoidance of physical activity [17]. This is of particular concern because it may adversely affect prognosis by reducing participation in cardiac rehabilitation and limiting daily physical activity, both of which constitute important elements of secondary cardiovascular prevention. Notably, avoidance of physical exertion has been reported in up to 37% of patients within 2 years following ICD implantation, and patients’ avoidance may increase the risk of arrhythmogenesis and potentially life-threatening ventricular arrhythmias [17,42,43]. This is most likely mediated by autonomic imbalance, with a shift toward sympathetic predominance and reduced heart rate variability, both of which are well-recognized proarrhythmic factors in patients experiencing high levels of anxiety and depression [43]. As a result, anxiety may contribute to an increased frequency of ICD therapies, which in turn reinforces fear of future shocks. Moreover, patients with higher baseline anxiety levels may perceive device shocks as more painful than other patients and may be at increased risk of recurrent shock-related distress [44,45]. In the context of anxiety assessment, it is important to distinguish between state anxiety and trait anxiety. State anxiety refers to a transient emotional response associated with a specific situation and a temporary sense of threat. By contrast, trait anxiety reflects a relatively stable personality disposition characterized by a tendency to perceive objectively non-threatening situations as threatening and to respond with disproportionately intense anxiety [46,47,48]. This distinction is clinically relevant, as trait anxiety may help identify patients at particularly high risk of persistent psychological distress following CIED implantation. Given these considerations, systematic assessment of anxiety in patients undergoing CIED implantation should constitute an important component of routine clinical care. At present, however, there is no single universally accepted tool dedicated specifically to anxiety assessment in this patient group.

Several validated instruments are available for anxiety assessment in patients with cardiovascular disease and CIEDs, each addressing different aspects of psychological distress. The Cardiac Anxiety Questionnaire (CAQ) particularly focuses on heart-related anxiety, the Hospital Anxiety and Depression Scale—Anxiety subscale (HADS-A) screens for generalized anxiety symptoms, and the State-Trait Anxiety Inventory (STAI) differentiates between state and trait anxiety, whereas Florida Shock Anxiety Scale (FSAS) specifically evaluates anxiety related to ICD shocks and device therapy (Table 2) [49,50,51,52,53]. This scale may be especially useful for monitoring shock-related anxiety during follow-up and for identifying patients who may benefit from targeted psychological intervention. The main anxiety assessment tools used in patients with cardiovascular disease and CIEDs are summarized in Table 2.

### 3.3. Depression

Depression is one of the most common psychiatric comorbidities in patients with cardiovascular disease and represents a major determinant of both clinical outcomes and quality of life. In patients with cardiovascular diseases, among those patients undergoing cardiac rehabilitation and those treated with CIEDs, the prevalence of depressive symptoms may reach up to 64%, particularly in the early phases of treatment [4,6,7,8]. From a clinical perspective, depression in cardiovascular patients is heterogeneous and may arise from multiple mechanisms. It may reflect a continuation of previously diagnosed affective disorders, develop as a psychological response, functional decline, or illness, or be associated with organic brain injury, including neurodegenerative processes or cerebrovascular disease [6,8]. In practice, these mechanisms often coexist, particularly in elderly and multimorbid patients. Depressive symptomatology can be broadly divided into somatic and cognitive–affective components. Somatic symptoms include fatigue, sleep disturbances, and reduced vitality, whereas cognitive–affective symptoms encompass negative self-perception, hopelessness, and pessimistic expectations regarding the future. Both domains have been associated with adverse outcomes; however, available evidence suggests that somatic symptoms may be more strongly associated with mortality in patients with cardiovascular disease. In patients with CIEDs, depression frequently coexists with anxiety and contributes to a substantial psychological burden. Available data suggest that the prevalence of depression is comparable between patients with ICDs and those with permanent pacemakers [24,54]. However, certain clinical factors appear to increase the risk of depressive symptoms, including atrial fibrillation, poor response to device therapy, and worsening heart failure, particularly with deterioration by at least one NYHA functional class [55]. Device-related factors also play an important role. A history of ICD shocks has been consistently identified as a significant predictor of depressive symptoms. Patients experiencing shocks—particularly repeated or inappropriate therapies—may develop or exhibit worsening depressive symptoms compared to those without such events. Moreover, even in patients without prior psychiatric history, ICD shocks may have a substantial negative psychological impact and have been associated with a several-fold increase in the risk of developing depressive symptoms [56,57]. Depressive symptoms present before device implantation may persist or worsen after the procedure, particularly in patients with ongoing heart failure symptoms or poor clinical response. Therefore, early identification of depression may improve risk stratification and optimize patient care. Among the available assessment tools, the Beck Depression Inventory, second edition (BDI-II), is one of the most commonly used instruments for evaluating depressive symptoms in both clinical practice and research settings [57,58]. Importantly, even incremental increases in depressive symptom burden appear to have prognostic significance. In patients with CRT or CRT-D devices, each one-point increase in BDI score has been associated with an approximately 5% increase in all-cause mortality risk [51]. Given the strong association between depression and adverse cardiovascular outcomes, routine screening for depressive symptoms should be considered an integral component of the care pathway in patients undergoing CIED implantation, both before and during follow-up [57].

### 3.4. Post-Traumatic Stress Disorders

Post-traumatic stress disorder (PTSD) represents an important yet often underrecognized psychiatric condition in patients with cardiovascular disease, particularly in those exposed to life-threatening cardiac events or invasive therapies [47]. Anxiety, depression, and PTSD frequently coexist, creating a burden that may significantly affect both quality of life and clinical outcomes. PTSD may occur following life-threatening cardiovascular events such as sudden cardiac arrest, malignant arrhythmias, or acute coronary syndromes, and in patients with ICDs may also be triggered by device therapies, particularly recurrent shocks, resulting in symptoms including hypervigilance, sleep disturbances, and persistent anxiety [47,59,60,61,62,63,64]. Several risk factors for the development of PTSD symptoms in ICD recipients have been identified. These include female sex, the presence of depressive symptoms, and post-traumatic dissociation, defined as a psychological disconnection from distressing experiences [59,63,64]. Additional risk factors include high baseline anxiety, Type D personality traits, and exposure to device therapies within the first months following implantation. Higher symptom severity has also been observed in patients with a history of myocardial infarction. PTSD may also develop following sudden cardiac arrest or hospitalization for severe cardiac events. The prevalence of PTSD symptoms in survivors of cardiac arrest has been reported to range from approximately 20% to 40%, with some studies demonstrating positive screening results in up to 30% of patients [65]. Among patients with ICDs, particularly those who have experienced shocks, reported prevalence rates range from 19% to 38%. Unlike classical PTSD, PTSD following sudden cardiac arrest or ICD therapy may develop despite limited or absent recollection of the triggering event, often presenting predominantly with hypervigilance, anxiety, and avoidance behaviors rather than intrusive memories or flashbacks [59,63,64,65]. Several tools may support the assessment of PTSD symptoms in patients with CIEDs. These include the PTSD Checklist for DSM-5 (PCL-5), the PTSD Checklist-Specific (PCL-S), and the Clinician-Administered PTSD Scale (CAPS-5) [66,67,68,69]. Their use may facilitate the early identification of patients at risk and enable timely intervention. Given the significant impact of PTSD on psychological well-being, functional status, and potentially cardiovascular outcomes, a multidisciplinary approach to care is essential. Close collaboration between cardiologists, psychiatrists, and psychologists may improve recognition and management of PTSD in patients with CIEDs and contribute to improved overall patient care.

## 4. Discussion

Mental health disorders and cognitive impairment represent a major and growing challenge in the aging population, with particularly high relevance in patients with cardiovascular disease. Large epidemiological studies have shown that nearly half of older adults experience at least one psychiatric disorder during their lifetime, with anxiety and mood disorders being the most prevalent [6,70]. This burden is substantially reflected in patients with cardiovascular disease, in whom psychosocial factors—including depression, anxiety, PTSD, and social isolation—are increasingly recognized as important determinants of clinical outcomes, as highlighted in the recent Polish and European consensus statements [4,71].

The integration of psychiatric and cognitive assessment into CIED care is also consistent with the emerging framework of behavioral cardiology, a discipline seeking to incorporate individual psychosocial profiles, such as personality traits, chronic stress, depression, and social isolation, into cardiovascular risk evaluation and treatment strategies. A recently described HEARTBEAT model is one example of such an approach, combining psychosocial profiling with conventional risk assessment within a multidisciplinary framework applicable to both primary and secondary cardiovascular prevention [72]. Although this model was not specifically developed for patients with CIEDs, its underlying principles are highly relevant to this population, in whom psychological and cognitive factors may influence treatment adherence, adaptation to therapy, and clinical outcomes.

Patients considered for, or already with, a CIED represent a population often characterized by advanced age, multimorbidity, and high psychological burden related both to the underlying disease and to device-related experiences [73]. Psychiatric and cognitive disorders are common in patients undergoing CIED implantation and remain insufficiently recognized in routine practice despite their potential impact on adherence, quality of life, and clinical outcomes. Data suggest that psychiatric conditions are frequently underdiagnosed and undertreated, with a substantial proportion of patients not receiving optimal treatment despite clinically significant symptoms [71]. From a clinical perspective, the most important question is not whether mental health assessment should be performed, but rather when and by whom it should be implemented.

First, it may be optimal to perform psychological and cognitive evaluation already during the pre-implantation assessment. At this stage, screening tools such as MMSE or MoCA (for cognitive function) and BDI-II or HADS (for mood disorders) may provide valuable information regarding the patient’s ability to provide informed consent, adhere to post-procedural recommendations, and cope with device therapy. In patients considered for ICD implantation, additional tools such as the FSAS may help identify individuals at high risk of shock-related anxiety. Second, reassessment should be performed during follow-up, particularly within the first months after implantation and after clinically significant events such as ICD shocks or HF decompensations. A follow-up evaluation at approximately 6–12 months appears reasonable, with additional assessments guided by clinical presentation or concerns raised by the patient or caregivers. Patients with ICDs or CRT-D devices require particular attention, given their higher risk of anxiety, depression, and PTSD, particularly following inappropriate shocks [47]. In terms of responsibility, initial screening may be effectively performed by cardiologists or other members of the cardiology team using brief, validated tools, as described above. However, patients with positive screening results or clinically significant symptoms should be referred for specialist evaluation by psychiatrists or psychologists. A collaborative, multidisciplinary model of care is likely to provide the greatest benefit, as psychiatrists or psychologists experienced in the management of cardiac patients may better address the specific psychological and cognitive challenges associated with CIED therapy; however, reluctance to seek psychiatric consultation should not preclude cardiologists from ascertaining that clinically significant depressive symptoms are recognized and appropriately addressed, including referral for specialist evaluation when indicated.

The clinical implications of cognitive impairment deserve particular emphasis. Beyond its impact on informed consent, cognitive dysfunction may compromise adherence to post-implantation recommendations, delay recognition of complications, and increase the risk of adverse events such as device infection or mechanical complications. Early identification allows the implementation of protective strategies, including caregiver involvement, individualized follow-up planning, and consideration of pro-cognitive treatment in selected patients at risk of further cognitive decline. Similarly, mood disorders and PTSD may significantly influence patient behavior and perception of device therapy. Avoidance of physical activity, fear of shocks, and reduced engagement in follow-up care may all contribute to worse outcomes. Importantly, psychological distress in ICD recipients may also extend beyond the direct experience of shocks and influence everyday behaviors, including excessive safety-seeking behaviors and altered use of modern technologies such as smartphones, reflecting persistent hypervigilance and anxiety related to arrhythmia risk [48]. Importantly, these conditions are potentially modifiable, and both pharmacological treatment—such as selective serotonin reuptake inhibitors—and psychotherapeutic interventions, particularly cognitive-behavioral therapy, have demonstrated benefit in this population [7,8]. The process of qualification for CIED implantation, especially in primary prevention ICD therapy, should incorporate shared decision-making. Psychiatric and cognitive status may significantly influence risk perception, treatment preferences, and the ability to understand long-term implications of therapy. In selected patients with severe or unstable psychiatric symptoms, postponement of device implantation until appropriate psychiatric treatment has been initiated and clinical stabilization achieved may be reasonable [44,57,63]. Particularly in patients with advanced dementia, limited life expectancy, or high expected burden of shocks, the appropriateness of device implantation should be carefully individualized, with active involvement of family members or caregivers. In this context, device selection may also be tailored to the patient’s psychological and cognitive profile, and the use of leadless pacemakers may be particularly advantageous in individuals at risk of device manipulation, poor adherence, or heightened device-related anxiety. However, as in patients with cognitive impairment, to date, limited data support this thesis in this cohort of subjects.

An often overlooked but clinically important aspect of long-term care is the discussion of ICD deactivation in the context of advanced disease or end-of-life care. In patients with progressive heart failure, dementia, or terminal illness, continuation of defibrillation therapy may result in painful shocks without meaningful prognostic benefit. Advanced care planning, including discussion of device management, should therefore be integrated into routine follow-up. Finally, mental health assessment should not be limited to patients alone. Partners and caregivers frequently experience psychological burden related to the presence of a cardiac device and the risk of sudden events. Their involvement in education and, when appropriate, psychological support may improve both patient outcomes and overall quality of care. In summary, psychiatric and cognitive disorders are common, clinically relevant, and potentially modifiable factors in patients undergoing CIED implantation. Their systematic assessment—both before and after implantation—should be considered an essential component of modern, patient-centered cardiac care.

### Limitations

Several limitations of this review should be acknowledged. First, the body of literature on psychiatric and cognitive disorders in CIED patients is characterized by heterogeneity with respect to study design, patient populations, follow-up duration, outcome definitions, and risk scores that are utilized, which limits the comparability of findings across studies. Second, the narrative approach, while appropriate for a broad overview of a clinically heterogeneous topic, may be prone to selection bias. Third, to date there are no prospective studies demonstrating that systematic psychological or cognitive screening before or after CIED implantation translates into measurable improvements in clinical outcomes. The recommendations made in this review are therefore based on pathophysiological reasoning, clinical experience, often indirect evidence, and opinion of authors, and should be interpreted with appropriate caution. Future prospective trials evaluating structured psychological assessment pathways in CIED patients may therefore validate these claims.

## 5. Conclusions

Mental health disorders and cognitive impairment are highly prevalent in patients considered for or treated with cardiac implantable electronic devices and significantly influence adherence, quality of life, and clinical outcomes. Although relatively infrequently performed in daily practice, a routine psychological and cognitive assessment should therefore be incorporated into standard care, particularly at the stage of pre-implantation evaluation and during follow-up, with special attention being paid to patients experiencing high-voltage ICD therapies. Initial screening may be performed by cardiology teams using simple tools, while patients with significant findings should be referred for specialist evaluation within a multidisciplinary framework.

## Figures and Tables

**Table 1:** Comparison between Mini-Mental State Examination (MMSE) and Montreal Cognitive Assessment (MoCA) scores for cognitive evaluation.

	Mini-Mental State Examination (MMSE)	Montreal Cognitive Assessment (MoCA)
**Purpose of assessment**	Initial dementia screening.	Initial screening for Mild Cognitive Impairment (MCI).
**Administration time**	5–10 min	10–15 min
**Sensitivity**	**Low**—Mild Cognitive Impairment (MCI). **High**: moderate and advanced dementia.	**High**—Mild Cognitive Impairment (MCI). **Moderate**: advanced dementia.
**Specificity**	**Higher**—lower false-positive rate in individuals without dementia.	**Lower**—higher false-positive rate.
**Cognitive domains assessed**	Orientation, registration, attention and calculation, recall, language, following commands.	Broader scope: Executive functions, episodic memory, visuospatial skills, attention, concentration, calculation, language.
**Clinical Utility**	Effective for rapid, routine assessment and monitoring of advanced dementia.	Preferred for early detection of MCI, which often presents as the initial symptom of dementia.

**Table 2:** Comparison of scores for evaluation of anxiety in patients with cardiac implantable electronic devices (CIED).

	Cardiac Anxiety Questionnaire (CAQ)	Hospital Anxiety and Depression Scale Anxiety Subscale (HADS-A)	State-Trait Anxiety Inventory (STAI)	Florida Shock Anxiety Scale (FSAS)
**Number of items**	**18**	**7**	**20 + 20**	**10**
**Scope**	Cardiac anxiety	Symptoms of general anxiety	State anxiety (transitory) vs. trait anxiety (stable personality characteristic)	Anxiety related to implantable device and shock delivery
**Specificity**	Scale assessing fear regarding cardiac symptoms, avoidance of symptoms, and hypervigilance toward physical sensations.	Scale assessing symptoms of anxiety and depression in somatic and clinical patients	A scale assessing anxiety both as an emotional state (temporary) and as a trait (stable predisposition to anxiety)	A scale assessing the level of anxiety regarding potential ICD shocks and situations that may trigger them.
**Clinical utility**	Diagnosis and assessment of cardiac anxiety.	Screening for generalized anxiety	In-depth assessment of anxiety in diagnostics and scientific research.	Assessment of anxiety in patients with an implantable cardioverter-defibrillator (ICD).
**Purpose of assessment**	Assessment of cardiac-related anxiety, particularly in patients with and without CIEDs.	Assessment of anxiety symptom severity independent of physical illness.	Measurement of anxiety levels both as a state and a personality trait.	Measurement of ICD shock-related anxiety and its impact on daily functioning.

## Data Availability

No new data were generated or analyzed in this study. All data supporting the findings of this review are derived from previously published articles cited in the reference list.
